# The Role of Adjuvant Treatment in Craniofacial Malignancy: A Critical Review

**DOI:** 10.3389/fonc.2020.01402

**Published:** 2020-08-07

**Authors:** Marton König, Terje Osnes, Øyvind Bruland, Kirsten Sundby Hall, Åse Bratland, Torstein R. Meling

**Affiliations:** ^1^Department of Neurosurgery, Oslo University Hospital, Oslo, Norway; ^2^Faculty of Medicine, Institute of Clinical Medicine, University of Oslo, Oslo, Norway; ^3^Department of Otorhinolaryngology, Head and Neck Surgery, Oslo University Hospital, Oslo, Norway; ^4^Department of Oncology, Oslo University Hospital, Oslo, Norway; ^5^Service de Neurochirurgie, Département des Neurosciences Cliniques, Hôpitaux Universitaires de Genève, Geneva, Switzerland; ^6^Faculty of Medicine, University of Geneva, Geneva, Switzerland

**Keywords:** skull base malignancies, adjuvant therapies, sinonasal cancer, olfactory, neuroblastoma, mucosal melanoma, malignant meningioma, soft tissue sarcoma

## Abstract

**Background:** Tumors originating from the craniofacial region usually present in a locally advanced stage with frequent involvement of adjacent sites and have a strong tendency for local recurrence in the absence of adjuvant therapy, even when the original surgical resection was presumed to be radical. In the past decades, several advances in the radiological diagnosis and treatment of craniofacial malignancies have been introduced. There are, however, no randomized trials that define the optimal multimodal treatment of these tumors because of their rarity as well as heterogeneity in both histology and site of origin. The aim of this study was to conduct a critical review of the role of adjuvant therapy in the treatment of craniofacial malignancy.

**Method:** We conducted a critical review of the past and contemporary literature available, focusing on adjuvant oncological treatments of the most common craniofacial malignancies.

**Results:** Preoperative radiotherapy can have a documented role in the treatment of olfactory neuroblastoma and soft tissue sarcoma, while preoperative chemotherapy can be advocated in the treatment of sinonasal undifferentiated carcinoma, neuroendocrine carcinoma, olfactory neuroblastoma, and craniofacial sarcoma (both soft-tissue and high-grade osteosarcoma). Postoperative radiotherapy has a well-established role in the treatment of most craniofacial malignancies. The role of postoperative chemotherapy is unclear in most histologies, but is commonly used during the treatment of well-selected cases of paranasal sinus carcinoma, olfactory neuroblastoma, mucosal melanoma, soft tissue sarcoma and high-grade craniofacial osteosarcoma.

**Discussion:** Alongside developments in surgery, there have also been improvements in diagnostics, radiotherapy, and chemotherapy. Implementation of novel radiation techniques allows delivery of higher radiation doses while minimizing irradiation-related morbidity. Better understanding of tumor biology allows the construction of more complex treatment strategies, incorporating adjuvant chemotherapy either pre- or postoperatively. In the era of personalized targeted therapy, rapid strides are being made to identify specific tumor-targets for use of novel biologic agents, with the potential to change current management paradigms.

## Introduction

The craniofacial region consists of several complex anatomic areas, closely related to the skull base, which pose surgical challenges for neurosurgeons and otorhinolaryngologists alike. Tumors originating from this region usually present in a locally advanced stage at diagnosis due to innocuous presenting symptoms and with frequent involvement of adjacent sites. In addition, there is a strong tendency for local recurrence in the absence of adjuvant therapy, even when the original surgical resection was presumed to be radical.

In the past decades, several advances in the radiological diagnosis and treatment of craniofacial malignancies have been introduced. Surgery in these locations may have dramatic functional and life-threatening consequences that sometimes prohibit radical surgical resections. However, novel surgical procedures and adjuvant modalities have made treatment feasible for malignancies previously considered impossible (1). There are no randomized trials that define the optimal multimodal treatment of malignancies of the craniofacial region because of the rarity of these tumors as well as their heterogeneity in both histology and site of origin.

This study aims to provide a critical review of the role of adjuvant therapy in the treatment of craniofacial malignancy.

### Craniofacial Malignancies

The term “craniofacial” refers to the parts of the head enclosing the brain and the face from the upper part of the maxilla, largely corresponding to the *suprastructure*. This anatomical region is affected by a variety of tumors with clinical, etiological, pathological, and genetic features distinct from other tumors in the head and neck. The skull base forms the floor of the cranial cavity and separates the brain from other facial structures. It can be subdivided into three regions: the anterior, middle, and posterior cranial fossae. The most important anatomic structure above the sinonasal region is the anterior skull base. This part of the skull base is aberrant to other regions of the cranial skeleton as it displays a unique configuration of an osseous cranial vault with depressions, ridges, and septa. The anterior skull base stretches between the posterior wall of the frontal sinus anteriorly, to the roof of the sphenoid sinus marked by the anterior clinoid processes and the planum sphenoidale, posteriorly. The lateral boundaries are formed by the frontal bone. The anatomical connection between the midface and neurocranium is formed by the maxilla, the nasoethmoidal complex, the palatal and vomerine bone, and the pterygoid process of the sphenoid. The jaw is constituted by two main parts: the maxilla (upper part) and the mandibula (lower part).

Although these tumors may have similar anatomical locations, they can have considerably different characteristics and clinical behavior. In addition, there is no universally accepted grading and staging system available for these tumors.

Malignancies in this region are rare and encompass a variety of cancers arising from different sites. While emphasizing their rarity, the most common and representative of these malignancies are sinonasal carcinoma, olfactory neuroblastoma, mucosal melanoma, soft-tissue sarcoma, malignant meningioma and malignant tumors of the bone and cartilage, such as osteosarcoma, chondrosarcoma, and chordoma.

## Adjuvant Therapies

Adjuvant therapies might be indicated when their efficacy—alone or in combination—is greater than their cumulative toxicity, depending on both patient-related and treatment-related factors (Table 1). They are usually administered with the intent of improving loco-regional control (i.e., enhancing the effect of surgery) with an impact on overall survival. Most commonly, these therapies include radiotherapy and/or chemotherapy administered pre and/or postoperatively (neoadjuvant and adjuvant therapy, respectively). In addition, novel therapies are currently being investigated, including agents selectively targeting extra- and intracellular signaling pathways, i.e., immune, hormone- and targeted therapies. At present, however, these therapies are mostly limited to therapy for overt metastatic disease (i.e., not as an adjuvant therapy), without primary surgical treatment.

**Table 1:** Factors influencing the efficacy of adjuvant therapies.

**Patient/tumor-related**	**Treatment-related**
Tumor histology, grade and stage	Treatment dose
Tumor oxygenation	Extent of body area treated
Mitotic fraction	Method of delivery
Genetic factors	Timing of delivery
Patient comorbidities	Combination of treatment
Medications and allergies	Pervious treatments
Age and performance status	Cumulative toxicity

Adjuvant therapies can be administered as a local or systemic treatment against the tumor (primary or recurrent), the resection site, local or regional lymph nodes, or to combat assumed distant subclinical/micrometastases.

Radiotherapy is used to achieve disease control at the tumor or lymph node site, and can be delivered using either using photons (X-rays) or heavy particles (proton beams or carbon ions). Fractionation is a commonly used process during the treatment of craniofacial malignancies, allowing maximal tumor cell death and minimal damage of nearby organs at risk (e.g., cranial nerves, eyes, brain).

When used as an adjuvant treatment, chemotherapy can be given as a radiosensitizer (i.e., rendering tumor cells more sensitive to radiation therapy by counteracting the radio-protective effect of tumor hypoxia). The two most commonly used agents are the cytotoxic agent cisplatin and the hypoxic modifier nimorazole.

However, chemotherapy can also be given as induction therapy or to minimize the subsequent risk of developing distant metastasis. For craniofacial malignancies, chemotherapy is usually administered as a part of a standardized regimen, and often as part of study protocols. The dosage is challenging; too low a dosage might be ineffective against the tumor, whereas too high dosage can lead to excessive—and sometimes intolerable—toxicity. All chemotherapy regimens require that the recipient is actually capable of undergoing treatment.

Targeted therapy interferes with specific molecules needed for carcinogenesis and tumor growth by targeting and blocking extracellular signals, rather than by blocking intracellular signals and interfering with all rapidly dividing cells (as traditional chemotherapy does) (2). A variety of molecular targets may be therapeutically relevant in some malignancies of the craniofacial region. In addition, identification of specific tumor markers may provide prognostic information that can be used to guide decision making and the selection of additional therapy. In some cases, participation in clinical trials that investigate immunotherapy and other novel approaches may be considered for patients with residual, recurrent, or metastatic disease (3–12).

### Toxicity in Oncological Treatment

Toxicity is influenced by patient-, treatment-, tumor- and physician-related factors (Table 2). Toxicity can also be treatment specific (Table 3). Radiation therapy may lead to several local and site-specific complications in the craniofacial region affecting the skin of the head, the eyes, and the brain. Such complications include epithelial surface damage, swelling, fibrosis, dryness, and cognitive decline. Chemotherapy, on the other hand, may lead to systemic complications such as immunosuppression, myelosuppression, gastrointestinal distress, organ damage, and fatigue. Both radiation therapy and chemotherapy may cause nausea and vomiting, hair loss, ototoxicity and neuropathy. In addition, secondary neoplasm is a possible long-term complication of both modalities. Toxicities might be cumulative through life, and the administration of adequate doses of adjuvant therapies might not be possible for the treatment of a secondary neoplasm, or—in the worst case—the therapy might not be available at all.

**Table 2:** Factors influencing the toxicity of adjuvant therapies.

**Patient-related**	**Treatment-related**	**Tumor-related**	**Physician-related**
Performance status	Ports used	Tumor site	Competence
Nutrition status	Energy selection	Tumor stage	Convenience
Hydration status	Dose	Tumor grade	Cost
Skin care	Beam modifying	Nodal status	Facilities
Oral hygiene	Fractionation		Multidisciplinary
Dental hygiene	Setup errors		
	Quality assurance		

**Table 3:** Treatment-specific toxicities.

**Radiotherapy**	**Chemotherapy**
Nausea and vomiting	Immune and myelosuppression
Epithelial surface damage	Gastrointestinal distress
Local swelling and fibrosis	Organ damage
Reduced wound healing	Fatigue, nausea, and vomiting
Hair loss	Neuropathy
Neuropathy and cognitive decline	Hair loss
Secondary neoplasm	Secondary neoplasm

### Timing of Adjuvant Therapies

Adjuvant therapy administered preoperatively (neoadjuvant) may shrink the primary tumor at the same time as instituting a treatment to avoid lymph node and/or visceral micrometastases developing into over metastases. Tumors with a low mitotic fraction experience a weaker response to radiation; in such cases, tumor control is often defined as lack of growth (and/or reduced cell density) rather than diminished size (13). In addition, preoperative therapy makes response-evaluation of the primary tumor feasible prior to surgery (i.e., induction chemotherapy). It is also advantageous that the blood supply to the tumor remains, i.e., not altered by surgery. However, neoadjuvant therapy may change both tumor and recipient characteristics, leading to difficulties regarding surgical treatment (Table 4).

**Table 4:** Potential benefits of adjuvant therapies pre and postoperatively.

**Preoperative therapy (neoadjuvant)**	**Postoperative therapy (adjuvant)**
Size reduction of primary tumor	Eradication of micro and macroscopic tumor rest
Eradication of micrometastatic disease	Reduced risk of recurrence and metastases

Postoperative adjuvant treatment has the potential to eradicate micro- or macroscopic tumor cells to improve survival, and to reduce the risk of both recurrence and metastases. In addition, features not available prior to surgery, such as complete histological evaluation, resection grade, and postoperative clinical status help further individualization of treatment, potentially increasing its efficacy and long-term survival for the patient (Table 4).

### The Role of Adjuvant Therapies

Defining the role of adjuvant therapies for craniofacial malignancies is challenging. The rarity and varied pathology of lesions in this anatomical region make it difficult to accrue large series of patients with uniform pathologies, and to date there are no randomized clinical trials to guide the treatment of patients with these malignancies. With only a few multi-institutional studies published, most reports in the literature are single-center series with limited numbers of patients and often short follow-up times, making results difficult to interpret and compare. In addition, treatment outcomes over long time-periods may be biased by medical and surgical developments. Selected publications providing relevant outcome measures are illustrated in Table 5.

**Table 5:** Selected publications reporting outcome measures after multimodal treatment of craniofacial malignancies.

**Publication**	**Histology**	**Treatment**	**5-year overall survival%**	**5-year progression free survival%**
Waldron et al. (15)	SCC, AC, SNUC	XRT + S	39	41
Paulino et al. (17)	SCC, AdCC, AC, MEC	XRT only	0	18
		S + XRT	52	50
Le et al. (20)	SCC, AC, AdCC, SNUC	XRT only	19	20
		S + XRT	46	56
Jansen et al. (19)	SCC, AC, AdCC, SNUC	XRT + S	60	65
		XRT only	9	47
Tran et al. (21)	AC, AdCC, MEC	XRT + S	n/a	18
		S only	n/a	62
		S + XRT	n/a	9
Tiwari et al. (22)	SCC, AC, AdCC, MEC, SNUC	S + XRT	64	n/a
		XRT + ChT	37 (2-yrs)	n/a
Fernström et al. (26)	SCC, AC, AdCC, SNUC, NEC, MEC	ChT + XRT + S	54	32
Dulguerov et al. (18)	SCC, AC, AdCC, MEC, SNUC	S only	79	n/a
		S + XRT	66	n/a
		XRT only	57	n/a
Amit et al. (36)	SNUC	IC + ChT + XRT	66	74 (with response to IC)
		IC + S + XRT	43	55 (with response to IC)
Yin et al. (51)	ONB	XRT+ S	91	91
		S + XRT	79	82
		XRT only	50	63
Chao et al. (55)	ONB	S + XRT	67	87
		XRT only	n/a	51
		S only	n/a	0
Dulguerov et al. (61)	ONB	S + XRT	65	n/a
		XRT + ChT	51	n/a
		S only	48	n/a
		S + XRT + ChT	47	n/a
		XRT only	37	n/a
De Bonnecaze et al. (50)	ONB	S + XRT	73	n/a
		S + XRT + ChT	64	n/a
		S only	58	n/a
		ChT + XRT	32	n/a
		XRT only	29	n/a
		ChT only	53	n/a
Amit et al. (67)	MA	S + ChT + XRT	47	n/a
		S + XRT	42	n/a
		S only	39	n/a
		ChT + S + XRT	27	n/a
Samstein et al. (71)	MA	S + XRT	n/a	59
		S only	n/a	35
Benlyazid et al. (136)	MA	S + XRT	28	29
		S only	46	27
Kaur et al. (180)	M WHO II	S + XRT	68	54
	M WHO III	S + XRT	56	48
Aghi et al. (173)	M WHO II	S + XRT	n/a	100
		S only	n/a	44
Mair et al. (181)	M WHO II	S + XRT	n/a	60
		S only	n/a	50
Dziuk et al. (167)	M WHO III	S + XRT	n/a	80
		S only	n/a	15
Jasnau et al. (88)	OS	ChT + S	75	52
		Cht + S + XRT	80 (2-yrs)	n/a 60
		S only	67	
Mucke et al. (90)	OS	ChT + S	67	n/a
		S only	42	n/a
Kassir et al. (94)	OS	S only	46	n/a
		S + XRT	20	n/a
		S + ChT	50	n/a
		S + XRT + ChT	67 (2-yrs)	n/a

*S, surgery; XRT, radiotherapy; ChT, chemotherapy; IC, induction chemotherapy; SCC, squamous cell carcinoma; AC, adenocarcinoma; AdCC, adenoid cystic carcinoma; MED, mucoepidermoid carcinoma; SNUC, sinonasal undifferentiated carcinoma; NEC, neuroendocrine carcinoma; ONB, olfactory neuroblastoma; MA, melanoma; M, meningioma; OS, osteosarcoma; n/a, not available*.

## Preoperative Therapy

### Paranasal Sinus Carcinoma

The role of preoperative radiotherapy is generally limited in patients with squamous cell carcinoma and adenocarcinoma of the paranasal sinuses as primary surgery provides a higher probability of radicality, lower complication rates, and also offers a precise histology with subsequent adjustment of postoperative radiation (14–17). Survival and local control in patients with advanced loco-regional tumors remain modest, with a meta-analysis showing an average 5-year survival of 51% (18). Radiation therapy alone or prior to salvage surgery should only be used when surgical resection is not feasible or is associated with unacceptable sequelae. Survival in such patients managed with primary radiation therapy with or without salvage surgery remains dismal (9–39%) (15, 17, 19, 20). If preoperative radiotherapy is used, a response-evaluation should be undertaken after 6–10 weeks to consider surgical resection of the tumor (14, 15, 17, 19–22).

The need to improve local control, increase survival and preserve organ function has prompted some centers to explore the addition of chemotherapy to standard treatment (23–26). Preoperative or induction chemotherapy can help to achieve operability in high-stage tumors, and can make radiation possible with less toxicity (23, 26, 27). The literature reports on a wide range of outcomes, and there are no definitive conclusions (28–30). Preoperative chemotherapy is usually not advised. There are concerns regarding possible disease progression during the treatment, and acquired cumulative toxicity leading patients being medically unfit for surgery (27). It can, however be considered in carefully selected cases where the tumor burden is so heavy that surgical resection or radiation is not possible without significant toxicity (27–29).

Sinonasal undifferentiated carcinoma and single-cell neuroendocrine carcinoma pose a unique therapeutic challenge to clinicians because of their aggressive biologic behavior, with a propensity (40–50%) for early invasion of vital structures such as the orbit, skull base, and brain, as well as a high risk of distant metastasis (20–30%) (31–35). In addition, these tumors are more chemo sensitive than other carcinomas in the same anatomical location (36, 37). Studies have documented the effect of platinum-based chemotherapy in these tumors, and intensive multimodal therapy is usually indicated, as oncological outcomes after open surgery remain poor (27, 36, 38, 39).

Patients with NUT-midline carcinoma—demonstrating loss of the ubiquitously expressed protein Integrase Interactor 1 (INI1; SMARCB1) —tend to present with large and locally advanced tumors; indeed, based on the previously reported series, most INI1-deficient sinonasal carcinomas are staged as T4 at the time of diagnosis. Experience suggests that these tumors respond well to neoadjuvant chemo-radiation (e.g., using a platinum based alkylating-like agent followed by radiation therapy) (40–45). Future treatments with agents that target the epigenetic machinery such as inhibitors against Enhancer of Zeste homolog 2 (EZH2) or histone deactylase may prove even more effective (46, 47).

### Olfactory Neuroblastoma

The benefit of radical surgical resection in terms of survival is well–documented (27, 48–50), however, the role of preoperative radiotherapy is unclear. According to Yin et al. (51) preoperative radiation therapy can provide a valuable complement to surgery. Experience from University of Virginia shows that patients who responded to preoperative adjuvant therapy (radiotherapy for low-stage tumor and chemotherapy plus radiotherapy for high-stage tumors, respectively), had significantly lower rates of disease-related mortality (52, 53). Although there is no clear evidence supporting the administration of preoperative adjuvant therapy for all patients, preoperative platinum-based chemotherapy can be advocated for patients with locally advanced disease (e.g., with intracranial and/or orbital invasion) (54–65).

While radical surgery followed by postoperative radiation is considered the standard of care in adults, a similar approach in children can lead to significant long-term morbidity. Preoperative chemotherapy based multimodal approach should be considered in children with advanced stage disease, as pediatric olfactory neuroblastoma is considered a chemosensitive disease. Radiation therapy is effective for local control but lower doses should be considered in children (66).

### Mucosal Melanoma

Preoperative radio-chemotherapy is generally not advocated for mucosal melanoma. However, radiotherapy may have a role when surgery is not appropriate or feasible (i.e., with palliative intent) (67–72).

### Soft-Tissue Sarcoma

Preoperative radiotherapy may allow some patients with soft-tissue sarcoma to undergo potentially less mutilating surgery, and can also contribute to a higher rate of local control in groups of patients with a dismal prognosis. Preoperative treatment may also permit lower radiation doses and smaller target volumes than postoperative radiotherapy (73–76).

Preoperative chemotherapy is usually recommended for most patients with rhabdomyosarcoma, whereas its role in the management of other histological soft-tissue sarcoma subtypes is unclear at present (74–80).

### Atypical and Malignant Meningioma

The use of radiotherapy or chemotherapy as a primary therapy with or without surgery is generally limited to patients medically unsuited for surgery or to those who have unresectable disease (81).

### Malignant Tumors of Bone and Cartilage

Although osteosarcoma is generally resistant to radiotherapy, proton-beam therapy may be useful in the treatment of chondrosarcomas and osteosarcomas that involve the skull base (82, 83). It can be particularly difficult to deliver sufficient radiation doses in cases of chondrosarcoma and chordoma due to nearby organs being at risk. Preoperative radiation therapy in these cases is generally not utilized (84–87).

Modern treatment regimens for classic osteosarcoma include preoperative chemotherapy to eradicate micrometastatic disease. Although its benefit in craniofacial osteosarcoma (CFOS) is controversial, preoperative chemotherapy has been associated with improved survival in patients with high-grade CFOS (88–97). Preoperative chemotherapy is not advocated in cases of chondrosarcoma and chordoma as these tumors are resistant (84, 86, 87).

## Postoperative Therapy

### Paranasal Sinus Carcinoma

Achieving radical resection in this anatomical location is challenging, and paranasal sinus carcinomas have a high tendency for local recurrence in the absence of postoperative radiotherapy (98). In general, adenocarcinoma (salivary gland type) is less sensitive to radiation therapy than squamous cell carcinoma. Salvage surgery may be warranted in recurrent cases, even when only close resection margins may be achieved. Postoperative radiotherapy is generally advised after non-radical surgery, or when radicality is questionable (14, 99–102). Adjuvant radiotherapy is widely used for stage T3 and T4 tumors, and has been effective in decreasing the incidence of local recurrence. However, there are no randomized trials or prospective comparisons, and the data in retrospective analyses are often based on older techniques (103–108). Commonly used conformal techniques include three-dimensional conformal radiotherapy (3D-CRT) and intensity-modulated radiotherapy (IMRT). Charged particle therapy may offer additional advantages for delivering maximal tumor doses while minimizing radiation to the retina and brain (104–106).

The role of postoperative radiotherapy in cases of stage T2 tumors is unclear, while some studies show no benefit, others show higher recurrence rates in the absence of radiotherapy, especially in cases of high-risk tumors, such as adenoid cystic carcinoma and undifferentiated carcinoma (108–111). In cases of squamous cell carcinoma with macroscopic or microscopic tumors after surgical treatment, concomitant hypoxic modification with the radiosensitizer nimorazol should be used (112).

Postoperative chemotherapy has been incorporated as a component of the multimodal therapy of paranasal sinus carcinoma in a variety of ways. Concomitant platinum-based chemotherapy (cisplatin and 5-FU) seems to have a positive effect on local control and survival and may have an additional benefit in cases of non-radical surgery, advanced-stage disease, and extracapsular tumor extension (24, 27–29, 108, 113–118).

The prognosis of patients with recurrent or metastatic head and neck squamous cell cancer is generally poor. Carefully selected patients with a good performance status and locally recurrent disease may benefit from salvage surgery and/or re-irradiation (119, 120).

Systemic therapy is indicated for most patients with metastatic or advanced recurrent squamous cell carcinoma of the head and neck. The choice of systemic regimen—preferably administered as a part of a study protocol—is influenced by multiple clinical factors, including patient comorbidities, performance status, previous therapy, and pathologic features (i.e., programmed death-ligand 1 [PD-L1] expression status). Treatment options include immunotherapy with PD-L1 checkpoint inhibitors (e.g., pembrolizumab and nivolumab), conventional cytotoxic chemotherapy (e.g., cisplatin and carboplatin) and molecularly targeted agents (e.g., epidermal growth factor receptor [EGFR] inhibitors cetuximab and panatimumab) (6–8, 121–130).

### Olfactory Neuroblastoma

Surgical resection followed by radiation therapy is the most widely used approach in cases of olfactory neuroblastoma. The results with this approach are illustrated by Dulguerov et al. who conducted a literature review and meta-analysis that included 390 patients from 26 studies published between 1990 and 2000 (61). For the 169 patients treated with a combination of surgery and radiation therapy, the reported 5-year survival rate was 65%. The reported 5-year survival rates for the 87 patients treated with surgery alone and the 49 patients treated with radiation alone were 48 and 37%, respectively. A similar added benefit of radiotherapy over surgery alone was shown for high-grade tumors in a Surveillance, Epidemiology, and End Results (SEER) study of 281 patients treated from 1973 to 2010 (65).

Several studies have documented that even patients with locally invasive tumors can achieve favorable long-term survival when surgical resection is followed by radiation (48, 54, 58–64, 131). The use of a combined-modality approach is particularly important for patients in whom disease extends beyond the paranasal sinuses or in whom surgical resection margins are positive (54, 59, 62).

The role of postoperative chemotherapy for olfactory neuroblastoma is unclear. Although several studies have shown improved results, the reason for these results (i.e., whether it is surgery, radiation, or chemotherapy) is unclear (37, 50, 53, 132–135). In general, adjuvant chemotherapy with cisplatin and etoposid is advocated in all cases of sinonasal cancer with small cell histology (27, 50).

The rarity of olfactory neuroblastoma, combined with the favorable prognosis following aggressive local and regional therapy, has resulted in only very limited experience for patients with disseminated disease. Chemotherapy appears to have activity in some patients (particularly cisplatin and etoposide), and newer molecularly targeted approaches (e.g., using sunitinib or by activating the sonic hedgehog pathway), may become an option as the biology of these tumors is better understood (10, 11).

### Mucosal Melanoma

Local recurrence occurs in 29–79% of cases with mucosal melanoma, despite aggressive surgery. Several series have reported an improvement in loco-regional control with postoperative radiotherapy; however, there is no verified impact on long-term survival and its role has not been established (68–72, 136–140).

There is only limited data available regarding the role and efficacy of postoperative chemotherapy in mucosal melanomas. A Phase II randomized trial of interferon vs. chemotherapy in Chinese patients with resected mucosal melanoma showed a superior effect of temozolamide and cisplatin, but these results require replication in a broader patient-population before postoperative chemotherapy can be considered a standard-of-care treatment for Western patients (141).

An understanding of the molecular pathogenesis of mucosal melanoma has provided important insights that are leading to the development of targeted therapies for specific subsets of patients with metastatic disease. Approximately 10% of mucosal melanomas harbor activating mutations in the BRAF gene and another 25% have somatic mutations or amplification of the tyrosine-protein kinase KIT (12, 142, 143). Several studies have reported durable tumor responses to KIT inhibition by imatinib, nilotimib, sorafenib, dasatinib, and sunitinib in patients with melanoma harboring KIT mutations (142, 144–148). In addition, checkpoint inhibitor immunotherapy (e.g., anti-CTLA4 and anti-PD-1 immunotherapy) has been shown to significantly prolong survival in some patients with cutaneous melanoma; however, additional investigation is necessary to clarify the role of these therapies in patients with mucosal melanoma (149, 150).

### Soft-Tissue Sarcoma

The benefit of postoperative radiation therapy for most histologic subtypes of soft-tissue sarcoma of the craniofacial region is controversial. Experience from the literature argues in favor of radiotherapy in cases of large tumors, high-grade tumors, and low-grade tumors with positive or close (<1 mm) resection margins (78, 151–158). Although radiotherapy for adults is commonly delivered through external beam radiation, for children with small, critically located tumors in the head and neck, intracavitary or interstitial implants (brachytherapy) may be an option (154, 159).

The indication for postoperative chemotherapy in soft-tissue sarcoma has to be determined individually and is only established in certain histotypes and high-grade sarcomas (77–79).

Given the limited efficacy of conventional cytotoxic chemotherapy, soft-tissue sarcoma remains fertile ground for the field of drug development. Clinical trials in a number of areas have shown promise in metastatic soft-tissue sarcoma, either as single agents or in combination with chemotherapy (160, 161).

### Atypical and Malignant Meningioma

Postoperative radiotherapy is advocated for all malignant meningiomas and subtotally resected atypical meningiomas of the craniofacial regions as complete surgical resection is generally difficult to achieve, and there is a high rate of both local recurrence and increased disease-specific mortality after non-radical surgery (81, 162–165). Data suggest that malignant meningiomas are associated with a recurrence rate 5 years after surgery of ~60–90% and a 5-year overall survival of 20–50% (164–169). Adjuvant radiotherapy appears to decrease the recurrence rate by approximately half and may increase 5-year survival to >50%. For patients who undergo incomplete resection or biopsy of an atypical meningioma, the rate of recurrence or progression ranges from 60 to 100% (170, 171). Adjuvant radiotherapy improves local control, aims to prevent further neurologic morbidity related to growth of the residual tumor, and may improve survival (172).

The role of adjuvant radiotherapy is unclear in atypical meningiomas with an apparent gross total resection. The potential benefits of radiotherapy are more closely balanced with its risks and side effects, and it is particularly important to assess individual patient preferences and tolerance for risk (162, 170, 173–179). Based on contemporary series, the reported recurrence rate after imaging-confirmed gross total resection in patients not treated with adjuvant radiotherapy is ~30–50% at a median of 5 years or less, with rates of failure trending higher with longer follow-up (173–175, 177). Most but not all observational studies suggest that adjuvant radiation therapy improves local control and progression-free survival after complete resection of an atypical meningioma (170–172, 177, 178, 180, 181). The impact of radiotherapy on overall survival is less clear, however, and most studies have included insufficient numbers of patients or length of follow-up to adequately assess this outcome. The potential benefits of radiation therapy should be weighed against the short- and long-term side effects and risks of this treatment method. Factors that increase the risk of side effects or delayed toxicities of radiation therapy include advanced age, low functional status, large treatment volume, and proximity of the radiation field to critical structures such as the optic pathways or pituitary gland.

The role of adjuvant postoperative chemotherapy for atypical and malignant meningiomas is unclear. Current guidelines of the National Comprehensive Cancer Network (NCCN) recommend three agents to treat patients with refractory and high-grade meningiomas: hydroxyurea, interferon-2B and sandostatin (long-acting release) (182, 183).

### Malignant Tumors of Bone and Cartilage

Postoperative radiotherapy is generally not advocated after radical, *en bloc* excision of CFOS. After non-radical surgery, re-excision should be performed. Radiotherapy (together with chemotherapy) is normally used for patients who are not candidates for re-excision or where the surgical margins remain positive after this attempt (83, 89, 184–186). In contrast, postoperative radiotherapy is suggested for most patients with chondrosarcomas and chordomas as complete resection of the tumor is difficult and recurrent tumors are associated with poorer prognosis (84, 85). Proton-beam therapy may be particularly useful as photon therapy is associated with a high rate of local failure and carries a significant risk of brainstem and cranial nerve damage (187–189).

Modern treatment regimens for osteosarcomas at non-head and neck sites generally include systemic cisplatin-based chemotherapy to eradicate occult micrometastatic disease. While chemotherapy (given either postoperatively or preoperatively) improves the prognosis of extremity osteosarcoma dramatically, its benefit in osteosarcoma of the head and neck is controversial.

Postoperative combination chemotherapy has a clear role in the management of high-grade CFOS; however, prospective data to support a benefit from adjuvant chemotherapy in head and neck osteosarcomas are lacking. In uncontrolled case series, the use of adjuvant or neoadjuvant chemotherapy has been associated with improved survival in patients with head and neck osteosarcomas in some (88, 90–93) but not all series (89, 190). Two meta-analyses on this subject reported conflicting conclusions, possibly due to incomplete information on the influence of surgical margin status (94, 95).

Whether patients with low-grade osteosarcomas benefit from chemotherapy is unclear. Most low-grade jaw osteosarcomas may be adequately treated with surgery alone, as long as clear margins can be achieved (191). The decision whether to pursue chemotherapy for very small high-grade and very large low-grade tumors must be individualized and made on a case-by-case basis (88, 89, 91–93, 95).

Postoperative chemotherapy has no role in the treatment of chondrosarcomas and chordomas, and it is hoped that novel therapeutics like targeted therapy will benefit these patients (2, 86, 87, 192–195). The relative lack of efficacy of conventional chemotherapy and the discovery of novel signaling pathways in several histologic subtypes of chondrosarcoma have prompted interest in molecular-targeted therapies (e.g., imatinib, dasatinib, sirolimus), particularly for chemotherapy-refractory non-operable or metastatic tumors (196–198). A variety of molecular targets may be relevant therapeutically in chordoma, including platelet-derived growth factor receptor (PDGFR), epidermal growth factor receptor (EGFR), vascular endothelial growth factor receptor (VEGFR), mammalian target of rapamycin (mTOR), and the *INI1* gene (2, 86, 87).

### Management of the Neck in Sinonasal Cancer

Postoperative irradiation (or lymph node dissection) of the neck is advocated for all patients with cervical lymph node involvement, while elective prophylactic treatment (in node negative patients) is controversial, and the optimal management in these cases is uncertain (54, 61, 65, 103, 199, 200). Radiotherapy is usually not necessary if there is N1 disease without extranodal extension, and neck dissection has been completed (201–203).

## Discussion

Alongside developments in surgery, there have also been improvements in diagnostics, radiotherapy, and chemotherapy. Implementation of routine 3D treatment planning and IMRT allows delivery of higher radiation doses to the tumor while minimizing morbidity caused by irradiation of normal structures. At the same time, a better understanding of tumor biology allows the construction of more complex treatment strategies that incorporate adjuvant chemotherapy either pre or postoperatively. In the era of personalized targeted therapy, rapid strides are being made to identify specific tumor targets for the use of novel biologic agents, with the potential to change current management paradigms.

Management decisions are complicated by the rarity of these entities and the resulting lack of consensus regarding the optimal treatment regimen. Most studies suffer from a small number of patients and inconsistent treatment strategies. Although there is agreement that multimodal therapy is needed, the optimal sequence and combination of treatment modalities are not known. Inclusion bias is common upon assessment on treatment outcomes, as patients with higher stage tumors are prone to be selected for combination therapy rather than surgery alone. In addition, reporting of survival function in the literature is not uniform, leading to difficulties with the comparison of results. In general, malignancies of the craniofacial region have a high tendency for local recurrence in the absence of adjuvant (postoperative) radiotherapy, even when the original resection was thought to be radical. Although there are no randomized trials, adjuvant radiotherapy is widely used and has been effective in decreasing the incidence of local recurrence (103). The effect of radiotherapy depends on tumor histology and is greatest in olfactory neuroblastoma, squamous cell carcinoma, and rhabdomyosarcoma, whereas the effect is less clear in adenocarcinoma and chondrosarcoma (204). Arguments for postoperative administration of radiotherapy (rather than preoperative) are as follows: a probably higher chance for local radicality; more precise evaluation of tumor volume and tumor margins; histology at primary surgery; and the possibility of more focused radiotherapy to reduce the danger of the dose affecting nearby organs at risk (205, 206). The use of radiotherapy alone or in combination with chemotherapy is generally limited to those who are medically unsuited for surgery or to patients with unresectable disease (207).

Advances in radiotherapy techniques have led to the development of highly conformal techniques that permit the delivery of therapeutic doses to the skull base while minimizing the dose to uninvolved vital structures (e.g., nerves, vessels, eyes). The most frequently used conformal techniques are 3D-CRT and IMRT (104, 208, 209). Charged particle irradiation (by proton beam or carbon ion) irradiation may offer additional advantages for delivering maximal tumor doses, while minimizing radiation to the retina and brain compared with photon-based therapy (105, 106, 210).

The role of chemotherapy in the treatment of craniofacial malignancies is unclear. Chemotherapy has been incorporated as a component of multimodality therapy with radiotherapy and/or surgery in a variety of ways; however, as there are no randomized trials, no definitive conclusions can be drawn about the impact of chemotherapy on outcomes.

A possible advantage of giving chemotherapy before loco-regional treatment (neoadjuvant) is more optimal drug delivery, permitting higher chemotherapy doses and dose intensities compared with chemotherapy given during or after local therapy. Possible disadvantages include a slow recovery from toxicity and, when the interplay between different modalities is less than optimal, delay of loco-regional treatment (still is the cornerstone of the intervention) may be fatally counterproductive (27).

Adjuvant chemoradiotherapy has been less studied. Craniofacial and sinonasal malignancies have generally not been included in trials evaluating the impact of chemotherapy as a radiosensitizer, and only limited experience has been gained from retrospective analyses (27).

**Figure 1:**
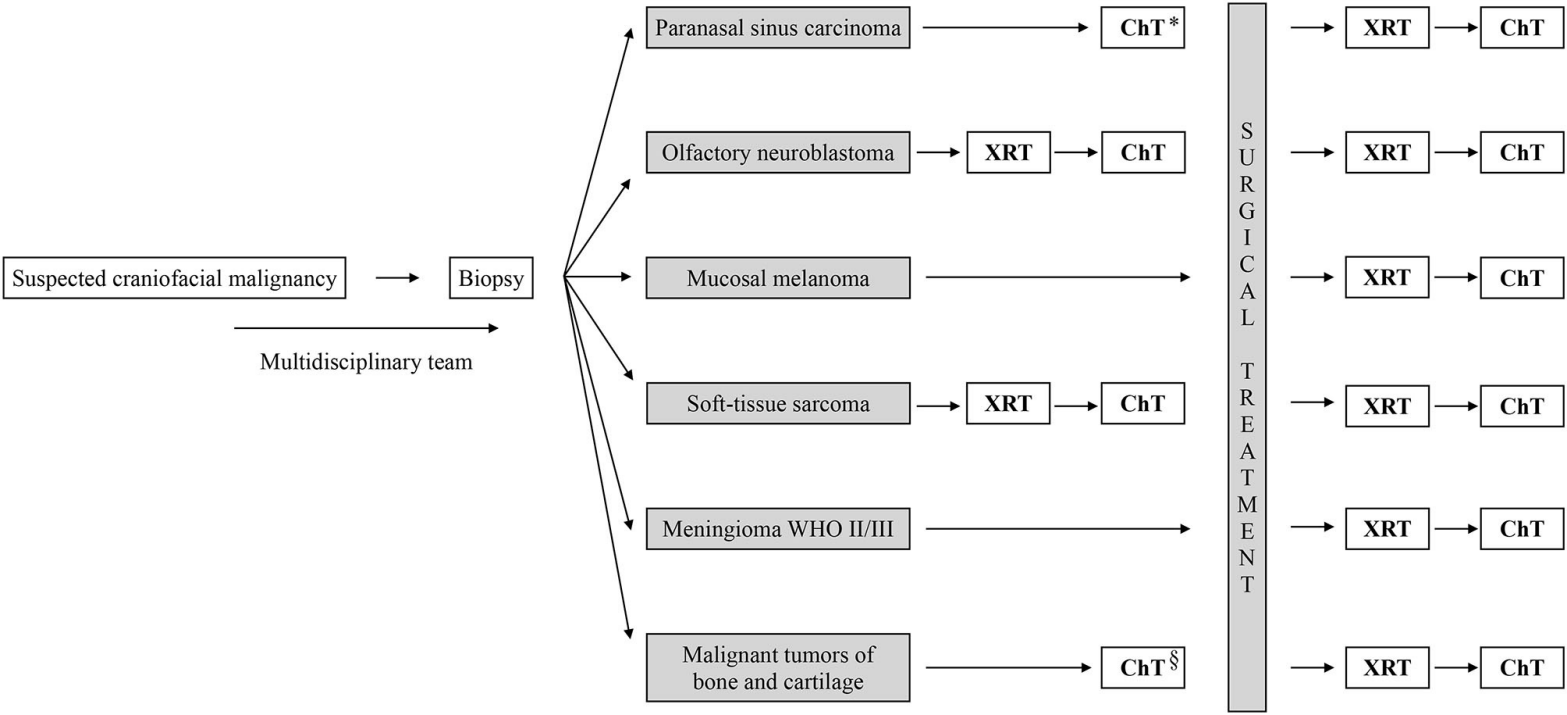
Summary work flow diagram for adjuvant treatment of craniofacial malignancies. *Sinonasal undifferentiated carcinoma, neuroendocrine carcinoma and NUT-midline carcinoma. ^§^High-grade osteosarcoma. XRT, radiotherpy; ChT, chemotherpy.

This review shows that preoperative radiotherapy can have a documented role in the treatment of olfactory neuroblastoma and soft-tissue sarcoma, while preoperative chemotherapy can be advocated in the treatment of sinonasal undifferentiated carcinoma, neuroendocrine carcinoma, olfactory neuroblastoma, and craniofacial sarcoma (both soft-tissue and high-grade osteosarcoma). Postoperative radiotherapy has a well-established role in the treatment of most craniofacial malignancies, apart from mucosal melanoma. The role of postoperative chemotherapy is unclear in most histologies but is commonly used during the treatment of well-selected cases of paranasal sinus carcinoma, olfactory neuroblastoma, mucosal melanoma, soft-tissue sarcoma, and high-grade craniofacial osteosarcoma (Figure 1).

## Author Contributions

MK has conceived of the idea of the presented review, carried out a critical review of the literature, and wrote the manuscript. TM has supervised the review process. ØB, KS, ÅB, and TM have contributed to the final version of the manuscript with critical evaluation, suggestions, and expertise. All authors contributed to the article and approved the submitted version.

## Conflict of Interest

The authors declare that the research was conducted in the absence of any commercial or financial relationships that could be construed as a potential conflict of interest.
